# Apatinib added when NSCLC patients get slow progression with EGFR‐TKI: A prospective, single‐arm study

**DOI:** 10.1002/cam4.6737

**Published:** 2023-11-30

**Authors:** Minghui Liu, Xin Li, Hongbing Zhang, Fan Ren, Jinghao Liu, Yongwen Li, Ming Dong, Honglin Zhao, Song Xu, Hongyu Liu, Jun Chen

**Affiliations:** ^1^ Department of Lung Cancer Surgery Tianjin Medical University General Hospital Tianjin People's Republic of China; ^2^ Tianjin Key Laboratory of Lung Cancer Metastasis and Tumor Microenvironment Tianjin Lung Cancer Institute, Tianjin Medical University General Hospital Tianjin China

**Keywords:** apatinib, circulating tumor DNA, EGFR‐TKI, NSCLC, safety

## Abstract

**Background:**

Epidermal growth factor receptor tyrosine kinase inhibitors (EGFR‐TKI) acquired resistance was an inevitably events in NSCLC treatment.

**Aims:**

Intending to overcome the acquired resistance of EGFR‐TKI.

**Materials & Methods:**

A clinical trial was, we enrolled 12 patients who were slowly progressing on first‐generation EGFR‐TKI, and added apatinib when the patients got slow progression.

**Results:**

Seven patients were included in the efficacy analysis. The median PFS2 of apatinib combined with EGFR‐TKI was 8.2 months (95% CI, 7.3 m‐NA), and the total PFS reached 20.9 months (95% CI, 17.3 m‐NA) when plus PFS1. All the adverse events were manageable. The median PFS was significantly longer for circulating tumor DNA (ctDNA)‐cleared patients (8.4 months; 95% CI, 8.2‐NA) than for those ctDNA not cleared (7.1 months; 95% CI, 6.9‐NA) (*p* = 0.0082).

**Discussion:**

The addition of apatinib did improve the duration of first‐generation EGFR‐TKI use, and the duration was better than the first‐line use of third‐generation EGFR‐TKI.

**Conclusion:**

The addition of apatinib when the patients got slow progression after initial EGFR‐TKI therapy may be a good treatment option and the side effects are controllable. It is possible to monitor treatment efficacy using ctDNA.

## 1 INTRODUCTION

The overall 5‐year survival rate of advanced lung cancer is as low as 18% in the United States.^1^ The use of epidermal growth factor receptor tyrosine kinase inhibitors (EGFR‐TKIs) in the clinic improved the survival prognosis of lung cancer patients. However, the inevitable problem is EGFR‐TKI resistance. Most first generation EGFR‐TKIs, such as gefitinib and erlotinib, result in drug resistance by about 12 months.

Many mechanisms underlying the first generation of EGFR‐TKI‐acquired resistance have been revealed, such as T790M gatekeeper EGFR mutations,^2^ MET amplification, STAT3 hyperactivation, activated STST3, PIK3CA, conversion to small‐cell lung cancer and dysregulation of the cell cycle.^3^ Osimertinib, a third generation EGFR‐TKI, was effective in patients with the T790M mutation, but most patients develop resistance within 6 months.^4^ Therefore, it remains a challenge to overcome acquired resistance to first‐generation targeted drugs in lung cancer treatment.

Apatinib, a novel VEGF receptor 2 TKI, is an anti‐angiogenic drug with promising antitumor activity and a well‐tolerated toxicities in several malignancies.^5^ In advanced EGFR‐mutant NSCLC, as first‐line treatment, apatinib plus gefitinib resulted in a PFS of 13.7 months compared with 10.2 months in the gefitinib group.^6^ Although the concurrent use of apatinib and EGFR‐TKI improves the PFS, we think that perhaps a sequential mode of adding apatinib can achieve a longer PFS. In our study, we explored the clinical effectiveness of adding apatinib when NSCLC patients got slow progression with EGFR‐TKI.

In clinical practice, CT imaging and serum carcinoembryonic antigen (CEA) are now commonly used to assess response to treatment in NSCLC,^7^, ^8^ yet these approaches do not fully reflect the molecular and pathologic changes that occur in the tumor during treatment. Circulating tumor DNA (ctDNA), mainly released by apoptotic and necrotic cancer cells, has also been demonstrated to be useful for monitoring the treatment efficacy of targeted therapy, chemotherapy, and immunotherapy in NSCLC.^9^, ^10^, ^11^

In this study, a prospective, single‐arm study was conducted to assess the efficacy and safety of apatinib combined with EGFR‐TKI on patients who got slow progression after first line EGFR‐TKI treatment. Meanwhile, we comprehensively analyzed ctDNA to dynamically monitor the efficacy of treatment.

## 2 PATIENTS AND METHODS

Patients newly diagnosed with primary lung cancer in the Department of Lung Cancer Surgery of the Tianjin Medical University General Hospital from July 2018 to March 2021 were recruited. A total of 12 NSCLC patients who got slow progression after first line EGFR‐TKI treatment were enrolled, then the patients took 250 mg of apatinib per day combined with the original EGFR‐TKI until disease progression or unacceptable toxicity. Slow progression is defined here as when CEA test and imaging examination were performed at 2–4 months' intervals, the abnormal CEA level increased on three consecutive tests compared to the CEA level of pre‐EGFR‐TKI treatment or target lesions relatively increases 0%–20% compared to the previous assessment according to RECIST 1.1. Peripheral blood was collected at baseline and 2–4 months' intervals after starting combination therapy for CEA and ctDNA detection. The Ethical review approval was obtained from Tianjin Medical University General Hospital for this study (ID number for IRB approval: IRB‐2018‐124‐01). Biological samples and images were acquired with the written informed consent of the patient. The detailed patient inclusion and exclusion criteria, treatments, study design, outcomes measurements and assessments, ctDNA testing by an 825‐gene panel of Genetron Co. Ltd. were described in Data S1.

## 3 RESULTS

### 3.1 Clinicopathological characteristics of patients at baseline

**FIGURE 1:**
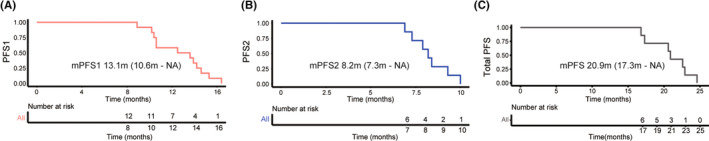
Kaplan–Meier analysis of PFS1 of 12 enrolled patients, PFS2 and total PFS of seven patients included in the efficacy analysis. (A) Kaplan–Meier analysis of PFS in 12 patients before slow progression for the first‐line EGFR‐TKI monotherapy (PFS1); (B) Kaplan–Meier analysis of PFS in seven patients for apatinib combined with EGFR‐TKI treatment (PFS2). (C) Kaplan–Meier analysis of total PFS of the seven patients. The median PFS and 95% CI are shown on the graph.

As shown in Table S1, the median age of 12 patients including seven (58.3%) females and five (41.7%) males were 66 years, ranging from 44 to 78 years. Most of the patients were non‐smokers (9/12, 75%). Two third (66.7%) patients were stage IIIB and the rest were stage IVa or IVb. All of them were diagnosed with adenocarcinoma. Two thirds patients (8/12) did not have metastasis, except for one patient with brain, two with bone and one with pleural metastasis. The first‐line EGFR‐TKI drugs patients received included gefitinib, icotinib and erlotinib. The median PFS1 was 13.1 months (95% CI, 10.6 m‐ NA), ranging from 8.9 to 16.4 months (Table S1; Figure 1A).

### 3.2 Treatments and efficacy

All 12 patients received oral apatinib (250 mg/day) combined with initial EGFR‐TKI of which dose were determined by the principal investigator. Seven of which were included in efficacy analysis who had at least one imaging follow‐up. Five patients were excluded because two of them lost follow‐up and three did not comply with the study protocol. All seven patients achieved SD. Until the last follow‐up in May 2021, all seven patients were still alive. The median PFS2 was 8.2 months (95% CI, 7.3 m‐NA), when plus the PFS1 for each patient, the total PFS reached 20.9 months (95% CI, 17.3 m‐NA) (Figure 1B,C).

### 3.3 Adverse events

Treatment‐related adverse events (AEs) were presented in Table S2. The most frequent AEs were grade 1/2 hypertension (6, 50%), hand‐and‐foot syndrome (4, 33.3%), anorexia (3, 25%), fecal occult blood (3, 25%), and proteinuria (2, 16.7%), There were no grade 3/4 AEs observed, and all of AEs were manageable.

### 3.4 Genetic profiles and KEGG pathway enrichment of baseline samples

Plasma were collected from 12 patients prior to apatinib combined with EGFR‐TKIs treatment as baseline samples. The genetic profiling showed that *EGFR* (25%), *FAT3* (17%), and *TP53* (17%) were the three most common mutated genes (Figure S2A). Next, KEGG pathway enrichment analysis showed that the mutated genes significantly enriched in FoxO and endocrine resistance signaling pathway (Figure S2B).

### 3.5 Serial ctDNA dynamically monitor the efficacy of combination treatment

**FIGURE 2:**
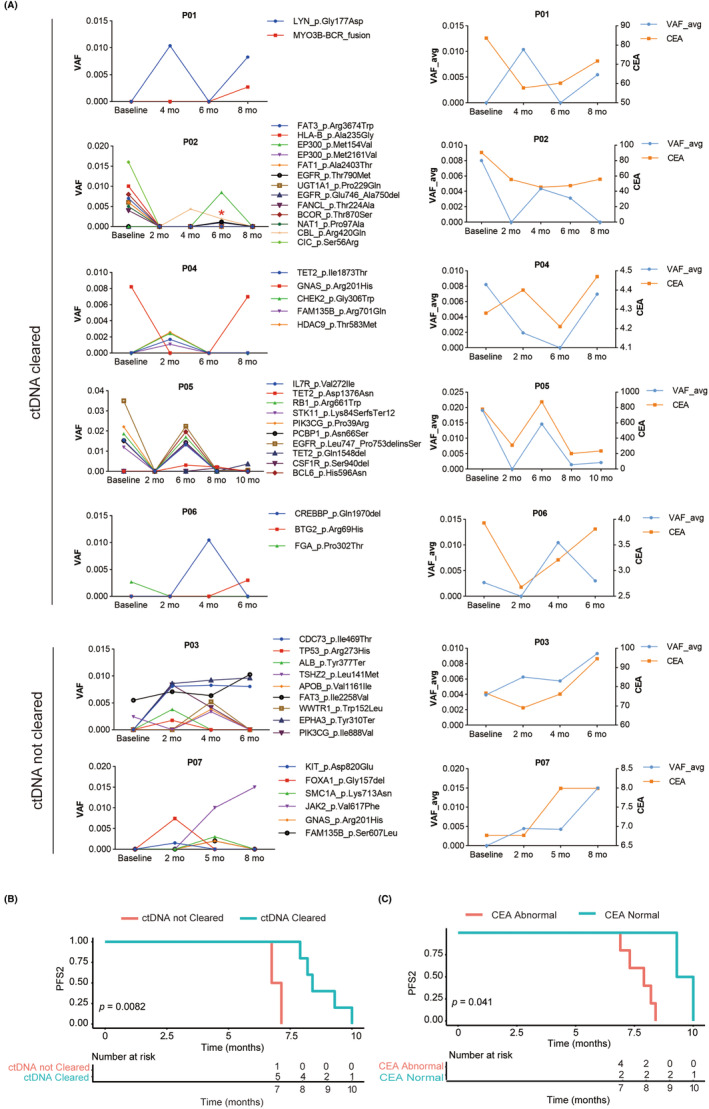
Dynamic Analysis of ctDNA VAF and CEA concentration of 7 patients included in the efficacy analysis. (A) Dynamic changes of ctDNA VAF for each gene (left panel) and the average ctDNA VAF and CEA concentration (ng/ml) (right panel) at 2–4 month’ intervals during apatinib and EGFR‐TKI combination therapy of each patient. The horizontal axis indicates the time since the combination therapy, and the vertical axis indicates the variant allele fraction of ctDNA or CEA concentration. A red asterisk indicates that an EGFR‐TKI resistant mutation T790M has been detected in patient P02. (B) Kaplan–Meier analysis of PFS stratified by ctDNA. (C) Kaplan–Meier analysis of PFS stratified by CEA. The *p* value was calculated using a two‐sided log‐rank test.

Plasma samples were collected from seven patients at 2–4 months intervals since starting combination therapy to dynamically monitor the changes of ctDNA and CEA. The dynamics of ctDNA variant allele fraction (VAF) was shown in Figure 2A, five patients: P01, P02, P04, P05, and P06, were found to have had at least once ctDNA cleared during monitoring. But for patients P07 and P03, ctDNA was always detectable. We found that the mPFS of ctDNA‐cleared patients (8.4 months, 95% CI, 8.2‐NA) was significantly longer than that of ctDNA not cleared patients (mPFS 7.1 months, 95% CI, 6.9‐NA, *p* = 0.0082) (Figure 2B). The ctDNA of patients P05 and P07 were continued to be monitored after disease progression and their ctDNA were both detected, particularly there was a large increase of ctDNA VAF for P07 (Figure 2A, Figure S2). The changes of ctDNA copy number per mL plasma showed almost the same as that of ctDNA VAF (Figure S3). For CEA monitoring, the concentrations of patients P04 and P06 were always within the normal range (<5 ng/mL), while the other patients were beyond the normal range (≥5 ng/mL). The mPFS in the normal CEA group (9.65 months; 95% CI, 9.3‐NA) was longer than that of the abnormal CEA group (7.9 months; 95% CI, 7.3‐NA; *p* = 0.041) (Figure 2C). For patient P07, after disease progression, ctDNA increased a lot while CEA remained stable, indicating that CEA is not a reliable marker reflecting the disease progression.

## 4 DISCUSSION

Our study aimed to extend the effective time of first‐line EGFR‐TKI by using sequential mode of adding apatinib. Previous studies WJTOG3405, NEJ002, and OPTIMAL indicated that the mPFS of first‐generation EGFR‐TKI as first‐line treatment for advanced EGFR‐mutated NSCLC was only 9–13 months.^12^, ^13^, ^14^ This limits the clinical application of EGFR‐TKI.

ARTEMIS (CTONG1509), NEJ026, RELAY, and ACTIVE (CTONG 1706) studies have demonstrated EGFR‐TKI combined with anti‐angiogenic drugs can prolong PFS compared to EGFR‐TKI alone.^6^, ^15^, ^16^, ^17^ Our results showed that adding apatinib prolonged the total mPFS to 20.9 months (95% CI, 17.3‐NA), longer than or comparable to the PFS of these previous studies. The difference between these previous studies and our study is the intervention time of anti‐angiogenic drugs. In these previous studies, the anti‐angiogenic drugs were used concurrently with EGFR‐TKI, but in our study, the anti‐angiogenic drug was added when patients got slow progression after EGFR‐TKI monotherapy, that is, a sequential mode. The results suggested although the effect of concurrent and sequential modes both extend the PFS of EGFR‐TKI, the sequential mode could reduce medical costs and perhaps reduce adverse effects of anti‐angiogenic drugs.

Using the third‐generation EGFR‐TKI as the first‐line treatment options is also an approach to extend the effective time of EGFR‐TKI. For example, the mPFS of osimertinib was 18.9 months,^18^ and aumolertinib was 19.3 months.^19^ It suggested the PFS of the third‐generation EGFR‐TKI as first‐line treatment options were comparable to the PFS in our study.

We considered the dose of apatinib in terms of both safety and efficacy. In Zhou's clinical trial, apatinib is administered at a dose of 500 mg or 750 mg daily.^20^ CTONG1706 study showed that 500 mg of apatinib daily combined with gefitinib results in more AEs with Grade ≥3 compared to other similar clinical trials.^6^ To determine the dose of apatinib, we first tried to treat some cases at a dose of 250 mg or 500 mg daily after obtaining informed consent, and found that 250 mg was as effective as 500 mg of apatinib when combined with EGFR‐TKI, but it was better tolerated by patients. Thus, we determined a dose of 250 mg. During our study, when the AE of patients was severe, we adjusted the dose of apatinib in two ways: 5 days a week or once every 2 days. If the AE does not abate, it is recommended to discontinue. Most patients can adapt to this dose adjustment.

In our study, we found a significant difference in PFS between the ctDNA‐cleared and not cleared groups (Figure 2A). Such responsive effect of ctDNA has also been reported in several studies. A study of ctDNA responsive to targeted therapy in NSCLC revealed that responders had nearly complete elimination of ctDNA, while non‐responders showed limited changes in ctDNA levels and significantly shorter PFS.^21^ Similar results were observed in both immunotherapy and chemotherapy efficacy monitoring.^11^, ^22^

The main limitation of this study is the small sample size, which may lead to analysis bias. But our study conveys valuable information about the function of apatinib in delaying EGFR‐TKI resistance and ctDNA profiling in monitoring efficacy. First, the addition of apatinib can prolong the effective time of the first‐generation EGFR‐TKI, providing a reference for solving the problem of TKI resistance in the clinic. Second, ctDNA may be a biomarker for monitoring treatment efficacy, and as a non‐invasive liquid biopsy, it may play a greater role in terms of efficacy monitoring in the future. To get a more reliable conclusion, further exploration of large‐scale, multi‐center clinical trials is needed.

## AUTHOR CONTRIBUTIONS

**Minghui Liu:** Data curation (lead); formal analysis (lead); project administration (supporting); validation (supporting); writing – original draft (lead); writing – review and editing (lead). **Xin Li:** Writing – original draft (supporting); writing – review and editing (supporting). **Hongbing Zhang:** Writing – original draft (equal); writing – review and editing (equal). **Fan Ren:** Resources (equal); software (equal). **Jinghao Liu:** Formal analysis (equal); software (equal). **Yongwen Li:** Data curation (equal); formal analysis (equal). **Ming Dong:** Investigation (equal); software (equal). **Honglin Zhao:** Software (equal); supervision (equal); validation (equal). **Song Xu:** Data curation (equal); investigation (equal); software (equal). **Hongyu Liu:** Formal analysis (equal); investigation (equal); validation (equal). **Jun Chen:** Project administration (lead); writing – original draft (supporting).

## FUNDING INFORMATION

This work was supported by the National Natural Science Foundation of China (82072595, 82172569, 81773207 and 61973232), Natural Science Foundation of Tianjin (19YFZCSY00040 and 19JCYBJC27000), Tianjin Key Medical Discipline (Specialty) Construction Project. Tianjin Health Science and Technology Project (ZC20179) and Tianjin Municipal Education Commission Natural Science Foundation (2019KJ202, 2021KJ221).

## CONFLICT OF INTEREST STATEMENT

All authors disclosed no commercial or financial relationships.

## ETHICS STATEMENT

The Ethical Review Committee of the Tianjin Medical University General Hospital approved the study protocol. All enrolled patients had read and signed informed consent. The study was conducted according to Good Clinical Practice and Helsinki Declaration principles. All biological samples, images and patients' information were obtained with patients' written informed consent.

## CLINICAL TRIAL REGISTRATION

This study was registered at http://www.chictr.org.cn/, the trial registration number (TRN) was ChiCTR1800019185 and the registration date was October 30th, 2018.

## Supporting information


Figure S1:

Figure S2:

Figure S3:

Table S1:

Table S2:

Data S1:

## Data Availability

The sequencing data analyzed in the study are available on the Genome Sequence Archive (GSA; http://bigd.big.ac.cn/gsa) with the accession number of HRA003997. If reasonable requests are made, the corresponding author will provide clinical data for this study.
