# Supplementary figures and images for: Apatinib added when NSCLC patients get slow progression with EGFR‐TKI: A prospective, single‐arm study
Source: Cancer Med. 2023 Nov 30;12(24):21735–41. doi: 10.1002/cam4.6737 (PMC10757148; doi:10.1002/cam4.6737)

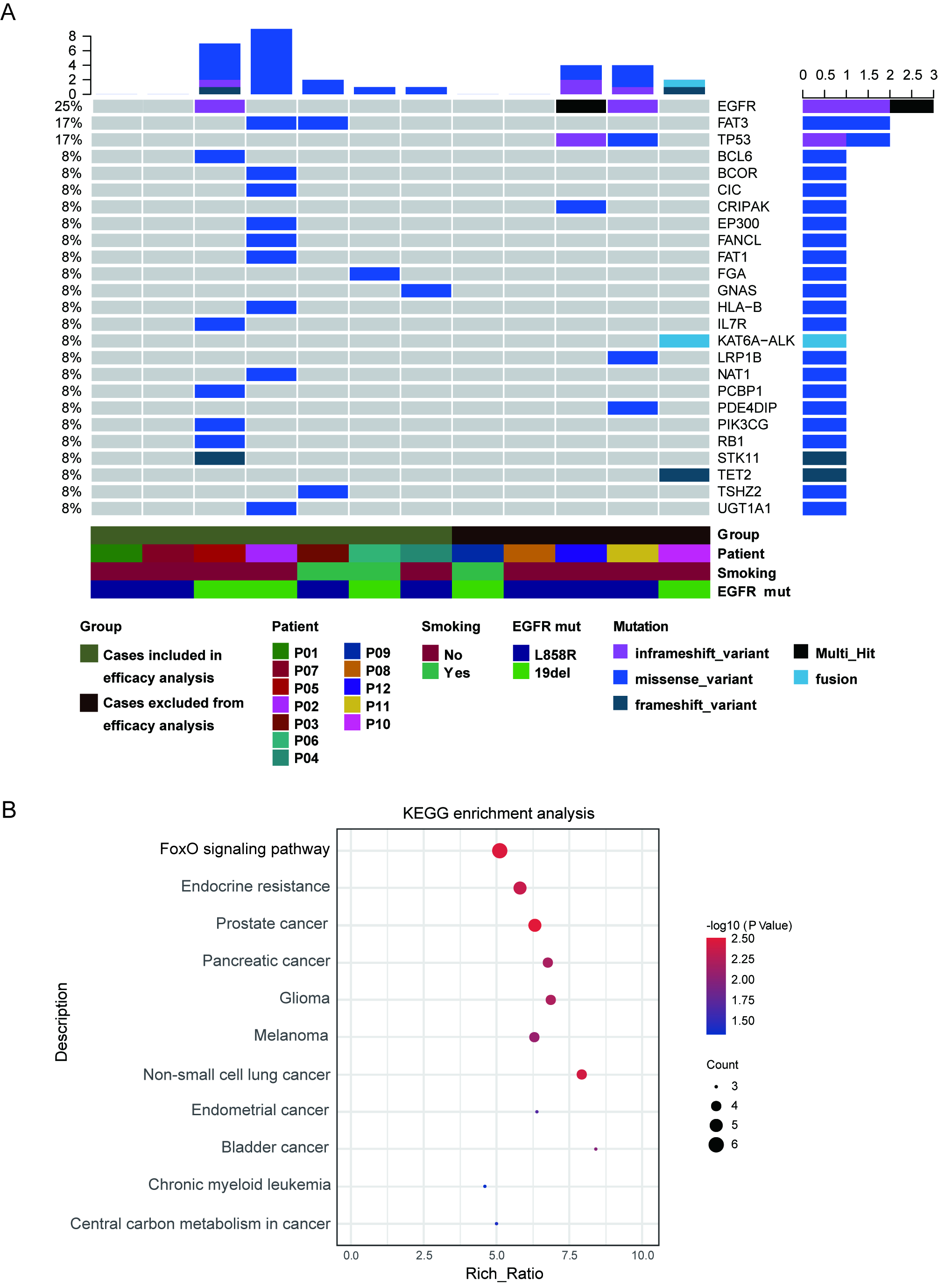

Supplement: Supplementary file 1 — Figure S1: [file CAM4-12-21735-s003.tif]

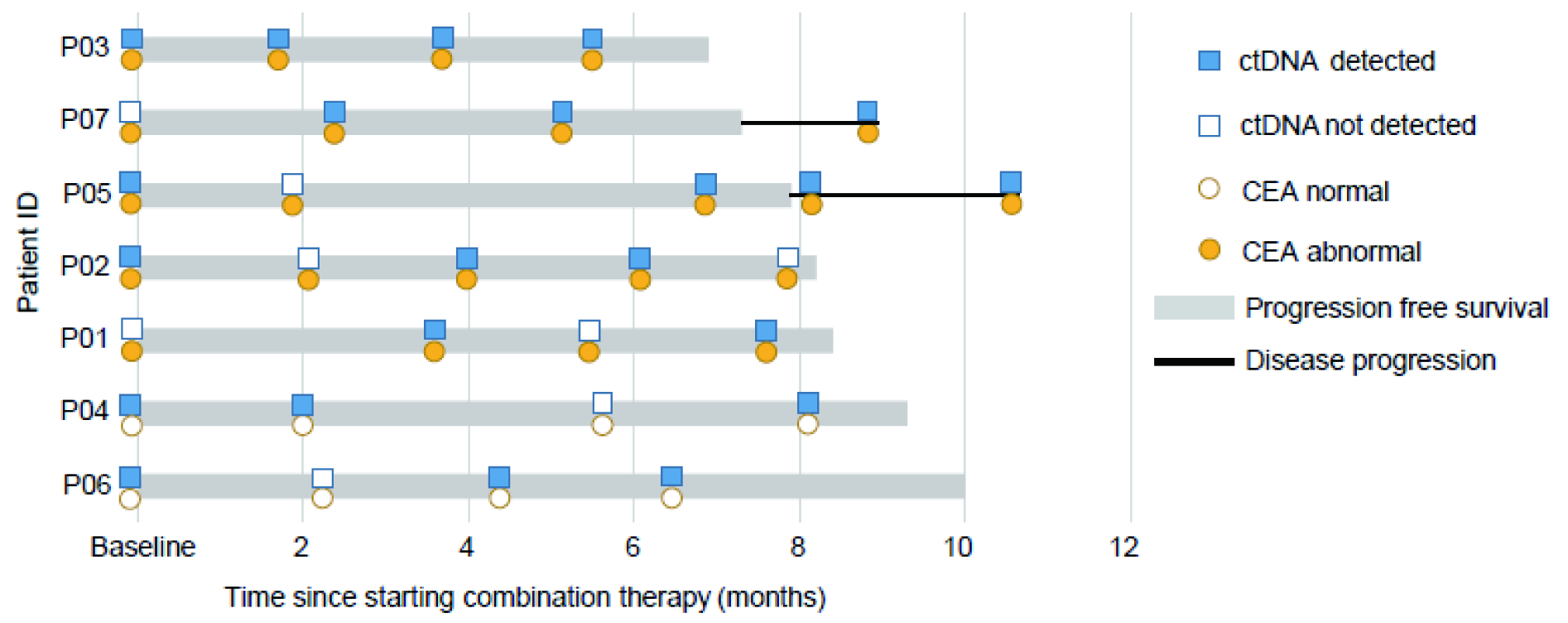

Supplement: Supplementary file 2 — Figure S2: [file CAM4-12-21735-s002.tif]

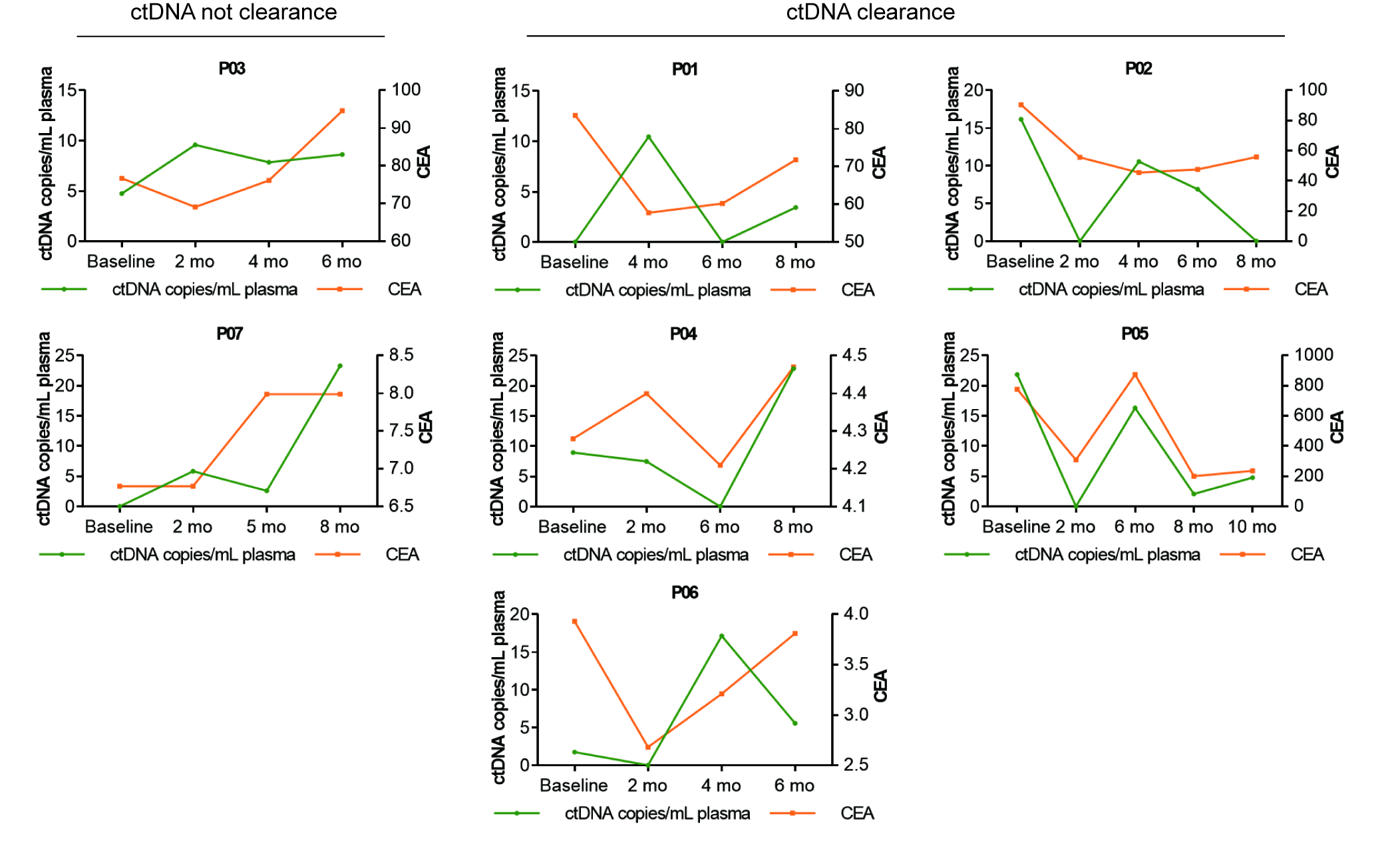

Supplement: Supplementary file 3 — Figure S3: [file CAM4-12-21735-s005.tif]
