# Supplementary material for: Apatinib added when NSCLC patients get slow progression with EGFR‐TKI: A prospective, single‐arm study
Source: Cancer Med. 2023 Nov 30;12(24):21735–41. doi: 10.1002/cam4.6737 (PMC10757148; doi:10.1002/cam4.6737)
Supplement: Supplementary file 6 — Data S1: [file CAM4-12-21735-s001.docx]

**Supplementary materials**

**PATIENTS AND METHODS**

### Patients

Patients with newly diagnosed primary lung cancer by pathology in the Department of Lung Cancer Surgery of the Tianjin Medical University General Hospital from July 2018 to March 2021 were recruited. The inclusion and exclusion criteria are previously described in details in the study protocol of this clinical study (1) . Describe briefly here, patients aged 18-80, diagnosed with stage IIIB or IV, non-squamous NSCLC, performance status of the Eastern Cooperative Oncology Group (ECOG): 0-2, expected survival beyond 12 weeks, and with initial EGFR 19 exon deletion or 21 exon (L858R) mutation and slow progression after first line EGFR-TKI were included. Slow progression is defined here as when CEA test and imaging examination were performed at 2-4 months’ intervals, the abnormal CEA level increased on 3 consecutive tests compared to the CEA level of pre-EGFR-TKI treatment or target lesions relatively increases 0%-20% compared to the previous assessment according to RECIST 1.1. Patients who had received interventional systemic therapy between cessation of first line EGFR-TKI and initiation of combination therapy were excluded. A total of 12 patients were included in the study based on the inclusion and exclusion criteria described above.

**Study Design**

This is a prospective, single-center, single-arm study. It was originally designed as a phase 2 clinical study but was stopped midway because COVID-19 resulted in too slow enrollment. The pre-treatment assessment included patient history, physical examination, complete blood count, serum chemistry, bronchoscopy, chest CT scan, and brain magnetic resonance imaging. The enrolled patients took 250 mg of apatinib per day for efficacy and safety reason on the basis of the original first-generation EGFR-TKI until disease progression occurred or there was unacceptable toxicity. The dose of EGFR-TKI including gefitinib, icotinib and erlotinib determined by the principal investigator. Patients were reviewed with Computed Tomography (CT) scan every two months. Peripheral blood was collected from patients at baseline and 2-4 months’ intervals after starting combination therapy for CEA and ctDNA detection. Ethical review approval was obtained from Tianjin Medical University General Hospital for this study (ID number for IRB approval: IRB-2018-124-01). Biological samples and images were acquired with the written informed consent of the patient.

### Outcome Measurements and Assessments

The co-primary endpoints were PFS2 and objective response rate evaluated by the investigators according to RECIST 1.1 and Choi criteria. PFS2 here means progression-free survival of apatinib combined EGFR-TKI therapy. Progression-free survival before slow progression for first-line EGFR-TKI monotherapy is defined as PFS1. The total PFS refers to PFS1 plus PFS2. In all eligible patients with at least one post-baseline assessment, the objective response rate was assessed. Secondary endpoints were overall survival, safety, and other factors. Safety analyses were performed on patients for whom complete safety data were available. Disease staging was assessed based on the TNM Classification of Malignant Tumors, 8th Edition. NGS for ctDNA was used to explore biomarkers for efficacy prediction and acquired resistance. The variant allele fraction (VAF) of ctDNA and ctDNA copy number per mL plasma were analyzed. ctDNA copy number per mL plasma here is calculated by the formula ‘Input DNA (ng)*1000 /3.3/ Plasma (mL)*VAF (mean)’. CEA normal means the concentration is less than 5 ng/ml, and abnormal means the concentration is higher than 5 ng/ml.

### ctDNA/gDNA Extraction

The gDNA of blood leukocytes and ctDNA of plasma were isolated using the AllPrep DNA/RNA Mini Kit (Qiagen 80204) and MagMaxTM Cell-Free DNA Isolation Kit (Thermo fisher A29319) respectively. Both gDNA and ctDNA were checked for fragment size, quality and total concentration by a 2200 Bioanalyzer (Agilent Technologies) to ensure their suitability for the following library construction.

### Next-Generation Sequencing and Data Analysis

A KAPA Hyper Prep kit (Kapa Biosystems) was used to construct the library of gDNA. To construct ctDNA library, first the 3' end was A-tailed, then 4bp random nucleotides were added to both ends of the ctDNA as a built-in tags and further adaptors were added by PCR amplification for HiSeq X Ten sequencing. The concentration of each library was determined by Qubit 3 (Thermo Fisher).

The gDNA and ctDNA libraries were enriched for regions of an in-house 825-genes panel of Genetron Co. Ltd. using a custom captured probe made by Agilent. The prepared libraries were first hybridized using the SureSelectXT Target Enrichment System (Agilent Technologies), then amplified with the P5/P7 primer. Finally, after quality checking with the 2200 Bioanalyzer and quantification with Qubit3 and a qPCR NGS library quantification kits (Agilent Technologies, Inc.), the libraries were sequenced on the Hiseq X10 platform (Illumina, SanDiego, CA).

The raw sequencing data were qualified by FASTQC (v0.11.9), and then the dual-index adapter sequences were demultiplexed and masked using Trimmomatic (v0.36)(2).The base pairs with Qphred ≤15 will be filtered out when setting 4 base pairs as a window. Sequencing reads were aligned with the GRCh37/hg19 genome using bwa (version 0.7.10)(3). The samtools (version 1.3.1, http://www.htslib.org/) and pindel (version 0.2.5b8, <http://gmt.genome.wustl.edu/packages/pindel/>) were used to call somatic SNVs and InDels, and only the SNV/InDel with paired mutation reads ≥ 4 and VAF > 0.1% were considered. The effects of the called variants were determined by Variant Effect Predictor (VEP, version92) (4). The filtering steps for variants include: first filter out false positive mutations using an internal false positive database of Genetron Co. Ltd., then filter out variants with frequencies >= 0.01 in the 1000 Genome Project database (https://www.internationalgenome.org/), and filter out the variants of which coding information are not 'exonic' or 'splicing', and finally retrieve hotspot mutations, etc. Artifacts associated with alignment and sequencing were filtered using IGV (5).

### Statistical Analysis

Progression-free survival (PFS) was defined as the time from treatment to tumor progression, death from any cause or the last follow-up. The cut-off time for follow-up was May 2021. All statistical analysis were performed on SPSS version 17.0 (SPSS Inc.). PFS rates were estimated using the Kaplan–Meier method. The dynamic change graph of ctDNA was created using GraphPad Prism 8 (GraphPad Software Inc., USA). A two sided P-value of 0.05 or less was regarded as statistically significant.

**References**

1. Li X, Liu M, Zhang H, Liu H, Chen J. Clinical study of apatinib combined with EGFR-TKI in the treatment of chronic progression after EGFR-TKI treatment in non-small cell lung cancer (ChiCTR1800019185). Thorac Cancer. 2020;11(3):819-26.

2. Bolger AM, Lohse M, Usadel B. Trimmomatic: a flexible trimmer for Illumina sequence data. Bioinformatics. 2014;30(15):2114-20.

3. Li H, Durbin R. Fast and accurate long-read alignment with Burrows-Wheeler transform. Bioinformatics. 2010;26(5):589-95.

4. McLaren W, Gil L, Hunt SE, Riat HS, Ritchie GR, Thormann A, et al. The Ensembl Variant Effect Predictor. Genome Biol. 2016;17(1):122.

5. Thorvaldsdóttir H, Robinson JT, Mesirov JP. Integrative Genomics Viewer (IGV): high-performance genomics data visualization and exploration. Brief Bioinform. 2013;14(2):178-92.
